# Kinases on Double Duty: A Review of UniProtKB Annotated Bifunctionality within the Kinome

**DOI:** 10.3390/biom12050685

**Published:** 2022-05-11

**Authors:** Aziz M. Rangwala, Victoria R. Mingione, George Georghiou, Markus A. Seeliger

**Affiliations:** Department of Pharmacological Sciences, Renaissance School of Medicine, Stony Brook University, Stony Brook, NY 11794, USA; victoria.mingione@stonybrook.edu (V.R.M.); geo.georghiou@gmail.com (G.G.)

**Keywords:** kinase, signal transduction, phosphorylation, cancer, inflammation, metabolism

## Abstract

Phosphorylation facilitates the regulation of all fundamental biological processes, which has triggered extensive research of protein kinases and their roles in human health and disease. In addition to their phosphotransferase activity, certain kinases have evolved to adopt additional catalytic functions, while others have completely lost all catalytic activity. We searched the Universal Protein Resource Knowledgebase (UniProtKB) database for bifunctional protein kinases and focused on kinases that are critical for bacterial and human cellular homeostasis. These kinases engage in diverse functional roles, ranging from environmental sensing and metabolic regulation to immune-host defense and cell cycle control. Herein, we describe their dual catalytic activities and how they contribute to disease pathogenesis.

## 1. Introduction

Protein phosphorylation regulates prokaryotic and eukaryotic cellular processes and signal transduction [1]. In humans, protein phosphorylation is mediated by 538 protein kinases, which comprise 3% of the human genome [2,3]. Protein kinases control metabolism, transcription, translation, cell cycle progression, cytoskeletal rearrangement, apoptosis, differentiation, intracellular communication, and homeostasis; thus, their dysregulation drives many human diseases. Development of small molecule kinase inhibitors has yielded 71 FDA-approved compounds used in the treatment of various cancers and inflammatory diseases [4,5].

Most FDA-approved kinase inhibitors bind to the highly conserved adenosine triphosphate (ATP)-binding pocket and prevent binding of substrate ATP. Surprisingly, some kinase targets have been found to retain or even increase their downstream signaling despite binding to ATP-competitive inhibitors. For example, certain BRAF inhibitors and kinase-dead BRAF mutants paradoxically activate downstream MEK phosphorylation by inducing dimerization and activation of uninhibited BRAF and CRAF [6,7,8]. The RAF family kinases exemplify the many noncatalytic functions of kinases, which extend to scaffolding, allosteric regulation, protein-DNA interactions, subcellular targeting, and beyond [9,10]. 

Protein kinases can exhibit catalytic activities other than phosphotransfer. For example, Ire1α is an evolutionarily conserved member of the unfolded protein response pathway (UPR) and contains both a protein kinase domain and an endoribonuclease domain. The UPR is a stress response pathway that responds to the accumulation of unfolded proteins in the endoplasmic reticulum (ER) by producing molecular chaperones and halting translation. Misfolded proteins induce Ire1α dimerization, which subsequently activates the ribonuclease domain (RNase) domain to splice transcription factor XBP1 and upregulate UPR response genes. Feldman and coworkers explored the allosteric relationship between Ire1α’s kinase and RNase domains by screening commercially available ATP-competitive kinase inhibitors and imidazopyrazine-based kinase-inhibiting RNase attenuators (KIRAs) [11]. Inhibitors of Ire1α kinase were found to bind and stabilize specific kinase domain conformations. Some kinase inhibitors promoted dimerization and activated the enzyme’s RNase activity, whereas the KIRAs stabilized monomeric Ire1α and inhibited both kinase and RNase activity. This work provides a proof-of-concept that ATP-competitive inhibitors can influence the non-phosphotransferase functions of their target kinases.

Bifunctional kinases such as Ire1α have the potential to reveal unique modes of allosteric regulation between different protein domains, as well as provide mechanistic insights into the functional benefits of having two signaling activities within the same enzyme. This knowledge can be further employed to guide drug discovery or drug repurposing campaigns toward finding unique strategies for specific and effective kinase inhibition. There may also be functional benefits to having two signaling activities on one enzyme, as is seen in other multi-domain enzyme complexes like fatty acid synthase.

In this review, we identify protein kinases documented with multiple catalytic activities in the UniProtKB database. We present a synopsis of our findings and highlight the bifunctionalities of bacterial and human kinases that are critical for cellular homeostasis.

## 2. Materials and Methods

We mined the UniProtKB (release version 2022_01), SwissProt, and TrEMBL databases [12] with a search strategy designed to identify Ser/Thr, Tyr, and dual-specificity kinases based on Enzyme Commission (EC) classification numbers. Although histidine kinases comprise much of the bacterial proteome, we restricted ourselves to only Ser/Thr and Tyr protein kinases, the critical mediators of signal transduction in humans.

The UniProtKB search algorithm employed in this study was as follows:

((ec:2.7.10.* OR ec:2.7.11.* OR ec:2.7.12.*) (ec:1.*.*.* OR ec:3.*.*.* OR ec:4.*.*.* OR ec:5.*.*.* OR ec:6.*.*.* OR ec:2.1.*.* OR ec:2.2.*.* OR ec:2.3.*.* OR ec:2.4.*.* OR ec:2.5.*.* OR ec:2.6.*.* OR ec:2.8.*.* OR ec:2.9.*.* OR ec:2.10.*.* OR ec:2.7.1.* OR ec:2.7.2.* OR ec:2.7.3.* OR ec:2.7.4.* OR ec:2.7.5.* OR ec:2.7.6.* OR ec:2.7.7.* OR ec:2.7.8.* OR ec:2.7.9.* OR ec:2.7.13.* OR ec:2.7.14.* OR ec:2.7.99.*)) OR (ec:2.7.10.* (ec:2.7.11.* OR ec:2.7.12.*)) OR (ec:2.7.11.* ec:2.7.12.*)

Our search returned 36,160 protein kinases with documented bifunctionality (Table S1). Of these kinases, 879 were Swiss-Prot Reviewed, with records extracted from the literature and curator-evaluated computational analysis. The remaining 35,281 kinases were TrEMBL Unreviewed, indicating that they have been computationally analyzed but still require full manual annotation by UniProtKB curators. We focused our efforts on the reviewed kinases to ensure that our selection contained verified bifunctional protein kinases (Table S2). We manually inspected all 879 entries to identify unique groups of homologous kinases.

## 3. Results

**Figure 1:**
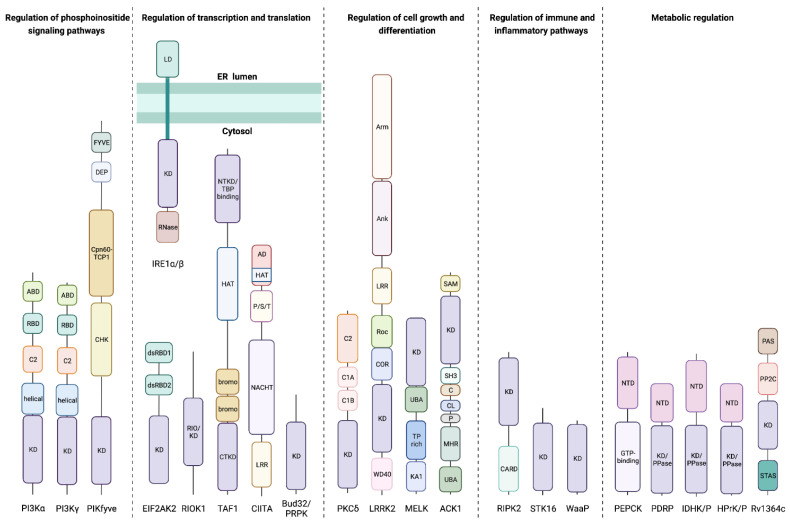
**Domain architecture of bifunctional kinases.** Representative structural domain schematic of 22 unique bifunctional kinases annotated in the UniProtKB database. Abbreviations denote the following: kinase domain (KD), Ras-binding domain (RBD), adaptor-binding domain (ABD), Cys-His-Lys homology domain (CHK), chaperonin-60–T-complex protein 1 subunit alpha domain (Cpn60-TCP1), disheveled–EGL10–pleckstrin domain (DEP), Fab 1–YOTB–Vac 1–EEA1 zinc finger domain (FYVE), endoribonuclease (RNase), luminal domain (LD), double-stranded RNA binding domain (dsRBD), C-terminal kinase domain (CTKD), N-terminal kinase domain (NTKD), TATA-box binding protein (TBP), histone acetyltransferase domain (HAT), bromodomain (bromo), leucine-rich repeat (LRR), GTP-binding domain (GTP), Pro-Ser-Thr rich domain (P/S/T), acidic domain (AD), β-transducin repeat (WD40), C-terminal of Roc domain (COR), Ras of complex G-domain (Roc), ankyrin domain (Ank), Armadillo domain (Arm), kinase-associated 1 domain (KA1), TP dipeptide-rich domain (TP rich), ubiquitin-associated region (UBA), Mig6 homology region (MHR), PPXY motif (P), clathrin-interacting region (CL), Cdc42/GTPase binding domain (C), Src homology 3 domain (SH3), sterile alpha motif (SAM), caspase activation/recruitment domain (CARD), N-terminal domain (NTD), kinase and phosphatase/phosphorylase/pyrophosphorylase domain (KD/PPase), Per-Arnt-Sim domain (PAS), PP2C-like phosphatase domain (PP2C).

Most of the kinases are dual-specificity Ser/Thr and Tyr protein kinases or protein kinases that predominantly exhibit other nucleotide triphosphatase activities. However, our search also returned examples of protein kinase activity paired with phosphatase, phosphorylase, endoribonuclease, acetyltransferase, carboxylyase, and sugar kinase catalytic functions. Given our interest in disease-relevant prokaryotic and eukaryotic kinases, we removed plant bifunctional kinases from the species *Arabidopsis thaliana* (22 kinases), *Oryza sativa* (2 kinases), and *Petunia integrifolia* (1 kinase), winnowing our initial 879 kinases down to 854 kinases. In addition, we removed two kinases found only in *Dictyostelium discoideum*, the bifunctional serine/threonine-protein kinase/NEDD4-like E3 ubiquitin-protein ligase and hybrid signal transduction histidine kinase G (dhkG). These kinases do not have homologs in humans, and their functions are inferred only from homology. Replicates across different bacterial strains made up most of the remaining 852 kinases, which we divided into 22 unique kinases (Table S3). General overviews of these kinases and their domain architectures are given in Table 1 and Figure 1, respectively.

## 4. Regulation of Phosphoinositide Signaling Pathways

### 4.1. Phosphatidylinositol 3-Kinases (PI3K)

PI3Ks are ubiquitously expressed intracellular **lipid kinases** that phosphorylate the 3′ hydroxyl groups of phosphatidylinositol and also harbor **Ser/Thr protein kinase** activity. These proteins regulate several critical cellular functions including cell survival, proliferation, motility, and vesicle trafficking [13,14]. PI3Ks are divided into three classes (class I, II, and III) based on their primary structure and substrate specificity. Class-I PI3Ks are further separated into subclasses IA (PI3Kα, PI3Kβ, PI3Kδ) and IB (PI3Kγ) based on their regulatory proteins and signaling pathway involvement.

PI3Ks function as heterodimers, consisting of a regulatory subunit and a catalytic subunit that converts the phosphatidylinositol second messenger PI(3,4)P2 (PIP2) to PI(3,4,5)P3 (PIP3) in response to extracellular stimuli on the activation of upstream receptor tyrosine kinases (RTKs) and G-protein coupled receptors (GPCRs) [15,16]. Conversion of PIP2 to PIP3 promotes the activation of downstream proteins AKT and mTOR [17]. PI3K activity is antagonized by the PTEN phosphatase, which hydrolyzes PIP3 to PIP2 [18]. As PIP3 plays a critical role in cell growth and replication, aberrant lipid kinase activity can promote oncogenic signaling. Class-I proteins have been recognized as promising drug targets due to their involvement in cancer and immune disease pathogenesis via the PI3K/mTOR pathway. The structural conservation of the ATP-binding pocket has complicated the development of isoform-specific inhibitors. Pan-PI3K inhibitors are active against all class-I isoforms and result in off-target toxicity and side effects. To date, there are five FDA-approved PI3K inhibitors (alpelisib, copanlisib, duvelisib, idelalisib, and umbralisib). While PI3K kinase inhibitors have been successfully applied to certain malignancies, their use and development has been hindered by poor drug tolerance and toxicity [19].

### 4.2. PI3Kα (PIK3CA)

*PIK3CA* encodes the phosphatidylinositol 3-kinase catalytic subunit alpha (PI3Kα), also known as p110α. PI3Kα is the predominant catalytic isoform for regulation of glucose homeostasis and is commonly mutated and amplified in a variety of cancers [20]. The catalytic p110α subunit is composed of five domains, including an adaptor binding domain (ABD) that binds to the Class-IA regulatory domains, a Ras binding domain (RBD), a C2 domain that binds to cell membranes, a helical domain with unknown function, and a catalytic kinase domain. PI3Kα activation is mediated by receptor tyrosine kinases (RTKs), Ras proteins, and additional small molecules including calmodulin [21,22].

The catalytic p110α subunit can couple to regulatory subunit p85α, the SH2 domain of which contains high-affinity binding sites to the phosphorylated tyrosine motif (pYXXM) found in the C-terminus of RTKs [23]. The RTK pYXXM motif disrupts interactions between the catalytic p110α subunit and regulatory p85α subunit and activates PI3Kα by releasing the inhibitory p85α SH2 domains from the catalytic p110α subunit. This release triggers a conformational change in PI3Kα that exposes the kinase domain and permits membrane binding [24]. It has been shown that p110α phosphorylates Ser608 of the p85α regulatory subunit and decreases catalytic activity in vitro [25]. Mutation of Ser608 to Ala or Glu reduced lipid kinase activity, as well as the interactions between the p110α catalytic subunit and p85α regulatory subunit. Thus, phosphorylation of Ser608 may reveal a mechanism for regulating activity by stabilizing the autoinhibited state.

Mutations in PI3Kα are frequently found in brain, breast, head and neck, endometrial, cervical, and gastric cancers [17,26,27]. Most mutations cause gain-of-function (GOF) and are found within the helical or kinase domains. It has been reported that GOF mutations in the helical domain require interactions with Ras-GTP, whereas kinase domain mutations require interactions with the p85 regulatory subunit [28]. Co-existing mutations in both domains have been found to synergistically increase the catalytic function and tumorigenic activity [29]. In addition, deletions in the C2 domain have been found to activate PI3K signaling while also increasing the sensitivity to PI3Kα inhibitors, suggesting that residues within the C2 domain are critical for for PI3Kα function [30]. There are several hotspot mutations in p110α that confer a gain of function, including helical domain mutations E545K and E542K and the catalytic domain mutation H1047R. Helical domain mutations E545K and E542K have been shown to suppress inhibition of p110α by the p85 regulatory subunit, which results in mutation-driven signaling that promotes glucose metabolism and cervical cancer cell proliferation [31]. Catalytic domain mutation H1047R has been shown to enhance interactions between p110α and the lipid membrane, thereby enhancing its lipid kinase activity and downstream signaling.

The importance of PI3Kα in disease has been well-established; in May 2019, alpelisib (Piqray™, Novartis, Morris County, NJ, USA) was the first PI3Kα inhibitor approved by the FDA for the treatment of breast cancer [32]. Alpelisib has specific activity for PI3Kα over other isoforms, despite their nearly identical active sites, and potently inhibits common mutations such as E545K and H1047R.

### 4.3. PI3Kγ (PIK3CG)

*PIK3CG* encodes for the phosphatidylinositol 3-kinase catalytic subunit gamma (PI3Kγ), also known as p110γ. PI3Kγ belongs to class-IB PI3Ks and has both **lipid and Ser/Thr protein kinase** activity. It regulates immune stimulation and suppression in inflammation and cancer [33]. The PI3Kγ catalytic subunit has five domains, consisting of a putative uncharacterized adaptor binding domain (ABD), a Ras-binding domain (RBD), a C2 domain for binding cell membranes, an α-helical domain, and a catalytic kinase domain [34,35]. PI3Kγ activation is primarily regulated through interactions with GPCRs, which occur through association of the PI3Kγ regulatory subunit with the G-protein βγ subunits [36,37]. It can also be activated through interaction between the PI3Kγ RBD domain and Ras GTPases [38]. In the absence of lipid membrane binding, PI3Kγ maintains an inactive conformation [39].

In contrast to PI3Kα, PI3Kγ is commonly upregulated or overexpressed in cancer rather than mutated; however, mutations have been identified in cancer patients [40,41]. Loss-of-function mutations in PI3Kγ cause severe immunodeficiency, highlighting PI3Kγ’s critical role in promoting appropriate adaptive immune responses [42]. PI3Kγ is also a driver of inflammatory and metabolic disorders including rheumatoid arthritis, atherosclerosis, lupus, obesity, and pulmonary fibrosis [43].

### 4.4. PIKfyve (PIKFYVE)

PIKfyve is a bifunctional **lipid kinase with Ser/Thr protein kinase** activity. PIKfyve and its enzymatic products regulate cellular processes including membrane trafficking, ion channel activity, cytoskeletal dynamics, nuclear transport, stress- and hormone-induced signaling, transcription, and cell cycle progression [44,45].

As a lipid kinase, it synthesizes phosphoinositides (PIs) PtdIns(3,5)P2 from PtdIns3P and PtdIns5P from PtdIns. Synthesis of PtdIns(3,5)P2 by PIKfyve is negatively regulated by the formation of a multi-protein complex with scaffolding regulator ArPIKfyve and phosphatase Sac3 [46]. The cryo-EM structure revealed that formation of this complex sterically hinders PIKfyve from accessing membrane-associated PIs [45]. Serine residue autophosphorylation of PIKfyve represses its own lipid kinase activity and simultaneously activates Sac3 lipid phosphatase activity to downregulate lipid product synthesis [45]. Sac3 is also a serine phosphatase that acts on PIKfyve to increase the lipid kinase activity [45]. PIKfyve kinase activity is required for Sac3 lipid phosphatase activity [45], meaning that its dual function is critical for maintaining the delicate balance of lipid homeostasis. However, given the presence of multiple phospho-sites on PIKfyve and its role as a target in multiple signaling pathways, how the delicate balance between the dual activities of PIKfyve and Sac3 is maintained remains unknown.

PIKfyve has four structured domains including a FYVE finger domain, which targets the protein to PtdIns3P-enriched endosome membranes, and a DEP domain with unknown function. The middle of the protein interacts with several binding partners and contains two domains, a Cpn60-TCP1 domain, which has sequence similarity to chaperonins, and a CHK homology region containing the conserved Cys, His, and Lys residues found in all PIKfyve orthologs. The PIKfyve catalytic domain controls all three of its catalytic functions (PtdIns(3,5)P2/PtdIns5P synthesis and protein phosphorylation) and contains a single ATP-binding site. Mutation of the catalytic Lys-1831 abrogates all three kinase activities [47]. Mutations in PIKfyve cause functional defects in endosomal sorting, leading to Francois-Neetens fleck corneal dystrophy [48].

## 5. Regulation of Transcription and Translation

### 5.1. Ire1α (ERN1)

Ire1α is a ubiquitously expressed transmembrane ER stress sensor that functions as a **Ser/Thr protein kinase and endoribonuclease**. It is the best-studied branch of the unfolded protein response (UPR), an integrated intracellular signal transduction pathway that is activated in response to the accumulation of unfolded proteins in the ER [49]. The UPR initially responds to ER stress by blocking translation and upregulating molecular chaperones and folding enzymes. Prolonged cell stress causes a switch to pro-apoptotic and pro-inflammatory signaling cascades. The UPR has become an attractive pathway for drug discovery efforts due to its involvement in cancer, inflammation, neurological disorders, diabetes, and ischemia-reperfusion injury [50].

Ire1α contains an N-terminal ER luminal domain bound to cytoplasmic Ser/Thr kinase and endoribonuclease domains through a transmembrane linker [51]. The accumulation of misfolded protein causes Ire1α to dimerize in a “back-to-back” orientation [52], resulting in the formation of high-order oligomers and release of the ER Hsp70 chaperone BiP from the inactive Ire1α monomer. Thus, Ire1α activation is facilitated by luminal domain dimerization and trans-autophosphorylation of the cytosolic kinase domains [53,54]. Phosphorylation of the Ire1α activation segment stabilizes the RNase domain, which excises a 26-nucleotide intron from XBP1 [52,55]. The two spliced exons are then ligated by RNA ligase RtcB to form XBP1s [56], which encode an essential transcription factor for downstream activation of UPR response genes [57,58]. 

### 5.2. Ire1β (ERN2)

Ire1β is a close paralog of Ire1α. It has dual **Ser/Thr protein kinase and endoribonuclease** activities and induces the unfolded protein response (UPR) through transcription factor XBP1 [55,58], albeit less effectively than Ire1α [59]. Like Ire1α, it contains separate kinase and endoribonuclease domains. Ire1β serves as a direct and dominant-negative suppressor of Ire1α, dampening the UPR and ER stress response in epithelial cells of the intestine and other mucosal surfaces [53,60].

### 5.3. Protein Kinase R (EIF2AK2)

The eukaryotic translation initiation factor 2α kinase 2 (EIF2AK2), also known as protein kinase R (PKR) and interferon-induced double-stranded RNA-activated protein kinase, is a dual-specificity **Ser/Thr and Tyr protein kinase**. It contains two dsRNA binding motifs (dsRBD) and a kinase domain. PKR has been extensively studied as a regulator of the integrated stress response to RNA and DNA viruses, working as both a translational repressor through phosphorylation of Ser-51 on EIF2α and as a transcriptional upregulator of innate immune pathway proteins including Iκβ and NF-κβ [61,62,63]. On DNA damage, PKR halts the G2-M cell cycle transition by phosphorylating Tyr-4 of CDK1, causing its ubiquitination and proteasomal degradation [64]. PKR is a critical signaling mediator of cell proliferation and apoptosis through its interactions with p38 MAP kinase, NF-κβ, and the insulin signaling pathways [63,65,66,67]. It is ubiquitously expressed in vertebrates [68] and is implicated in cancer, neurodegeneration, inflammation, aging, and metabolic disorders [69,70].

The most widely used PKR inhibitor is C16, an imidazo-oxindole inhibitor with an in vitro IC_50_ of 210 nM; however, less potent inhibitors including 2-aminopurine have also been developed [70]. As an ATP-competitive inhibitor, C16 inhibits both the Ser/Thr and Tyr kinase functions of PKR and has been investigated for use in memory enhancement [71], neurodegeneration [72], inflammation [73], and metabolic disorders [74].

### 5.4. RIOK1 (RIOK1)

RIOK1 is a **Ser/Thr protein kinase and ATPase** in the Rio (right open reading frame) family of atypical protein kinases. In humans, there are three Rio subfamilies: Rio1 and Rio2 are conserved across all kingdoms of life, while Rio3 is only found in multicellular eukaryotes [75]. Specifically, RIOK1 functions in processes essential for cell proliferation, cell cycle progression, and chromosome maintenance. Yeast and human RIOK1 kinases are essential for the biogenesis of small ribosomal subunits, and depletion of this enzyme causes cell cycle arrest in yeast. Although RIOK1 is classified as a Ser/Thr kinase [76], it predominantly acts as an ATPase and regulates pre-40S ribosomal subunit association [77]. 

The RIO kinase domain adopts the conserved bilobal kinase domain structure but lacks an activation loop and substrate recognition sites. It instead contains a C-terminal RIO-kinase specific αR helix and a flexible loop between β3 and helix αC, which together allow it to accommodate a phospho-Asp substrate and serve as an ATPase [78]. At this time, there are no specific RIOK1 inhibitors. However, RNAi-mediated knockdown of RIOK1 reduced cell proliferation in RAS-driven cancer cell lines [79], making it an alluring target for drug discovery efforts.

### 5.5. TATA-Box Binding Protein Associated Factor 1/Transcription Initiation Factor TFIID Subunit 1 (TAF1)

TATA-box binding protein associated factor 1 (TAF1) is a **Ser/Thr protein kinase and histone acetyltransferase** with ubiquitin-activation and -conjugation activities [80]. TAF1 is the largest subunit of TFIID and binds core promoter sequences and transcriptional regulators. TFIID, which initiates RNA-polymerase II-dependent transcription, is the core scaffold for the TFIID basal transcription factor complex [81,82]. TFIID is comprised of the evolutionarily conserved TATA binding protein (TBP) and multiple TBP-associated factors (TAFs).

TAF1 is a bipartite kinase composed of N- and C-terminal kinase domains (NTK and CTK) and a centrally located histone acetyltransferase domain (HAT) [83]. Between the HAT and CTK domains are two bromodomains that selectively bind to multiply acetylated histone H4 peptides [84]. TAF1 acetylates histones H3 and H4 [85], and TAF1-mediated acetylation of H3 is required for Sp1 activation of cyclin D1 transcription and G1 to S-phase cell cycle progression [86]. Both TAF1 kinase domains are necessary for Ser phosphorylation of the TFIIF subunit transcription factor RAP74 and initiation of the RNA polymerase II transcription complex assembly [83,87]. TAF1 also phosphorylates tumor suppressor p53 on Thr-55, resulting in Mdm2-mediated p53 degradation and progression through G1 of the cell cycle [88]. Interaction between retinoblastoma protein Rb and TAF1 inhibits its kinase activity, which suggests that TAF1 may play a role in tumor suppression [89]. Mutations to the TAF1 gene cause X-linked dystonia parkinsonism and X-linked syndromic intellectual development disorder-33 [90,91]; however, the specific mechanisms by which TAF1 mutations drive these diseases are unknown. 

### 5.6. MHC Class II Transactivator (CIITA)

MHC class-II transactivator (CIITA) is a **Ser/Thr protein kinase and histone acetyltransferase** that functions as a transcriptional coactivator and as the master regulator of major histone compatibility complex (MHC) class-II gene expression [92]. CIITA is a functional homolog of TAF1 and uses multiple mechanisms to activate transcription initiation and elongation [93,94]. CIITA phosphorylates the C-terminal of TAF7, a component of the TFIID promoter complex, and Ser-36 of histone H2B [95]. Its acetyltransferase domain is required for de novo transcription of MHC class-II genes and enhanced transcription of MHC class-I genes [96]. Autophosphorylation of CIITA markedly increases its acetyltransferase activity, which suggests that the acetyltransferase domain is regulated by CIITA’s intrinsic kinase activity [95].

CIITA is the only documented transcription factor in the nucleotide-binding oligomerization (NOD) family and contains a conserved tripartite structure consisting of a variable N-terminal effector-binding domain (EBD), a central NOD domain, and a C-terminal region with a variable number of leucine-rich repeats [93]. Its N-terminal acidic domain for transcriptional transactivation contains its histone acetyltransferase domain (HAT), followed by a proline-, serine-, and threonine-rich domain (P/S/T domain) with multiple phosphorylation sites [97]. The central nucleotide-binding domain (NACHT domain) acts as a kinase domain, and in conjunction with the C-terminal leucine-rich repeats, it affects self-association and nuclear import [98,99]. Mutations in the *CIITA* gene cause severe immunodeficiency syndrome, also known as bare lymphocyte syndrome [100]. 

### 5.7. p53-Related Protein Kinase (TP53RK) 

The p53-related protein kinase (PRPK) and its homologs Bud32/Kae1 are **Ser/Thr protein kinases and ATPases** present in eukaryotes including yeast, humans, and pathogenic fungi including *Candida albicans*, *Coccidioides immitis*, and *Cryptococcus neoformans*. PRPK is one component of the highly conserved EKC/KEOPS complex (endopeptidase-like and kinase associated to transcribed chromatin/kinase, endopeptidase, and other proteins of small size). The EKC/KEOPS complex is required for the universal threonyl carbamoyl adenosine (t6a) tRNA modification, which is found in all tRNAs that pair with ANN codons where N is any of the four bases [101]. The EKC/KEOPS complex and t6a modification are critical for life, with remarkable sequence conservation from archaea to mammals [102,103].

PRPK maintains the conserved human kinase domain architecture including the regulatory and catalytic spines, a DFG motif, and a salt bridge between the invariant lysine in β3 and the invariant glutamate in helix αC [104]. There is high functional conservation between PRPK and its yeast homolog Bud32 [105]. Bud32′s regulatory partner Kae1 (OSGEP in humans) switches its intrinsic kinase activity to ATPase activity, which is required for EKC/KEOPS complex function [106]. Kae1 binds to the C-terminal lobe of Bud32 while Cgi121 (TPRKB in humans) binds to the N-terminal lobe, with the catalytic residues Lys-52, Asp-161, and Asp-182 interacting between the two lobes [107]. Based on the similarity between Bud32 and the Rio2 atypical kinase, it is hypothesized that its ATPase activity is required for the dissociation of tRNA from the complex.

Mutations in PRPK and the EKC/KEOPS complex cause Galloway-Mowat syndrome, an autosomal recessive disease characterized by a combination of early-onset nephrotic syndrome and microcephaly with brain anomalies [108]. PRPK is phosphorylated at Ser-250 by AKT [109] and is a promising therapeutic target in colon adenocarcinomas, cutaneous squamous carcinomas [108], and multiple myeloma [110].

## 6. Regulation of Cell Growth and Differentiation

### 6.1. Protein Kinase C δ (PRKCD)

Protein kinase C delta type (PKCδ) is a dual-specificity **Ser/Thr and Tyr protein kinase**. It is a member of the protein kinase C (PKC) superfamily and regulates neutrophil and endothelial cell proinflammatory signaling through Ser/Thr/Tyr phosphorylation of a myriad of substrates [111,112]. Thus, it serves as a critical regulator of the inflammatory response in cancer, diabetes, heart disease, sepsis, and autoimmune disease [112]. PKCδ contains an N-terminal Ca^2+^-sensing domain, a kinase domain, and an AGC-kinase family C-terminal regulatory tail that stabilizes the kinase domain and serves as a docking surface for regulatory proteins [113].

Unlike other PKC isoforms, PKCδ is uniquely regulated by phosphorylation of tyrosine residues within the regulatory domain (Tyr-52, -64, -155, -187), hinge region (Tyr-311, -332), and catalytic domain (Tyr-505, -512, -523). Tyr phosphorylation within the catalytic domain increases PKCδ activity, whereas Tyr phosphorylation within the regulatory domain primarily influences downstream signaling [112]. Phosphorylation of regulatory domain Tyr-155 and hinge region Tyr-311 are both important for PKCδ-mediated proinflammatory signaling and the initiation of cytotoxic and apoptotic pathways [112,114]. Tyr-155 phosphorylation regulates gene expression and apoptosis, whereas Tyr-311 phosphorylation causes a conformational change that exposes the PKCδ caspase cleavage site [115]. Mutations of Tyr-155 increase cell proliferation in response to its substrate phorbol 12-myristate 13-acetate (PMA). In addition to regulating kinase activity, tyrosine phosphorylation also amplifies nuclear localization of PKCδ during the genotoxic stress response and induces apoptosis [115]. 

### 6.2. Leucine-Rich Repeat Ser/Thr Protein Kinase 2 (LRRK2)

Leucine-rich repeat serine/threonine-protein kinase 2 (LRRK2) is a **Ser/Thr protein kinase and GTPase** involved in neuronal plasticity [116], autophagy [117], and ciliogenesis [118]. LRRK2 is a multi-domain protein that belongs to the Roco family of G-proteins. This protein family contains a central Ras of complex proteins (Roc) G-domain with an adjacent C-terminal of Roc (COR) dimerization domain. LRRK2 also contains N-terminal armadillo, ankyrin, and leucine-rich repeat (LRR) scaffold domains, a kinase domain, and a C-terminal WD40 scaffold [119]. The Roc domain is a functional GTPase [120] responsible for activation and regulation of the kinase domain on binding to guanine nucleotides [121]. In the cytosol, GTP-bound LRRK2 is primarily monomeric and exhibits low kinase activity, whereas at the membrane it is dimeric and active [122]. The cytosolic localization of LRKK2 is stabilized by phosphorylation-dependent interactions between its N-terminal segment and the ubiquitous regulatory protein 14-3-3 [123]. LRRK2 primarily acts as a Ser/Thr kinase through phosphorylation of Rab GTPases, which are master regulators of intracellular vesicle trafficking and membrane transport [118]. GTP-bound Rab proteins bind to the N-terminus of LRRK2 and recruit it to the membrane, where it undergoes a multi-step hydrolysis cycle and results in dimeric GDP-bound LRRK2 [122]. LRRK2 dimerization at the membrane is mediated by its Roc/COR domains and is critical for GTP hydrolysis and achieving maximal kinase activity. On LRRK2 activation, it phosphorylates GTP-bound substrates. The interplay between GTPase activity and kinase activity reveals that these activities are clearly linked.

Mutations in LRRK2 are the most common genetic cause of Parkinson’s disease (PD) [124,125]. LRRK2 phosphorylates amyloid precursor protein at Thr-668 and cause loss of dopaminergic neurons [126,127] in PD. It also impairs proteasomal degradation of Tau and results in the accumulation of high-molecular-weight Tau neurofibrillary tangles, linking LRRK2 to dementia in PD and Alzheimer’s disease [128]. Interestingly, pathogenic mutations are found in both the kinase domain (G2019S, I2020T) and the Roc/COR GTPase domains (R1441C/G/H, Y1699C), suggesting that both enzymatic activities are crucial for PD pathogenesis [129,130,131]. The roles of LRKK2 in disease pathogenesis have stimulated the development of potent and selective inhibitors, such as third-generation ATP-competitive inhibitors MLi-2 and PF-06685360 [132,133]. Although inhibitors of LRRK2 have been identified, the lack of safety and side effects of these ATP-competitive compounds remain a challenge. Since the GTPase and kinase functions of LRRK2 depend on one another, its GTPase activity may be critical for pathogenicity. Thus, recent progress has been made toward developing allosteric LRRK2 inhibitors that mainly act by interfering with its GTPase activity rather than inhibiting kinase activity directly.

### 6.3. Maternal Embryonic Leucine Zipper Kinase (MELK)

The maternal embryonic leucine zipper kinase (MELK) is a dual-specificity **Ser/Thr and Tyr protein kinase** [134] from the adenosine monophosphate-activated protein kinase (AMPK)-related family. This protein family is highly upregulated in breast [135], brain [136], colorectal, gastric, and ovarian cancers [137]. MELK is predominantly a Ser/Thr kinase with broad substrate specificity and only phosphorylates tyrosine autocatalytically [134]. It is composed of a kinase domain followed by a ubiquitin-associated region, an autoinhibitory region, and a kinase-associated-1 domain. MELK mediates cell cycle progression through phosphorylation of CDC25B, promoting its localization to the centrosome and spindle poles during mitosis [138]. High MELK expression is associated with aggressive undifferentiated tumors; however, there is conflicting evidence regarding its role as an oncogenic driver [139]. 

### 6.4. ACK1 (TNK2)

Activated Cdc42 kinase 1 (ACK1) is a dual-specificity **non-receptor Tyr and Ser/Thr protein kinase** that functions as a specific effector kinase for the GTP-bound form of Cdc42. ACK1 is involved in cell survival, migration, spreading, growth, proliferation, and epigenetic regulation. It has eight distinct domains: a sterile alpha motif (SAM), a catalytic kinase domain, an SH3 domain, a Cdc42/GTPase binding domain, a clathrin interacting region, a PPXY motif, a Mig6 homology region (MHR) or EGFR binding domain, and a ubiquitin-association domain. The ACK1 multi-domain architecture facilitates its localization to different cellular compartments and association with binding partners [140]. 

ACK1 acts as a dual-specificity kinase toward Wiskott-Aldrich syndrome protein (WASP), an effector protein of Cdc42 that plays an integral role in actin filament formation. ACK1 phosphorylates WASP on Ser-242 and Tyr-256; Ser-242 phosphorylation was found to enhance WASP-mediated actin polymerization in cell lysates [141]. In addition, ACK1 acts as a protein tyrosine kinase toward AKT, a critical mediator of cell survival through inhibition of pro-apoptotic proteins. ACK1 directly phosphorylates AKT on the evolutionarily conserved kinase domain residue Tyr-176 [142]. Phosphorylation of AKT Tyr-176 localizes it to the plasma membrane and promotes Ser/Thr phosphorylation that leads to its activation [142].

ACK1 promotes tumorigenesis and metastasis in prostate cancer and tamoxifen-resistant breast cancer [143,144,145,146], and it is also implicated in systemic lupus erythematosus [147]. To identify potent and selective inhibitors of Ack1 kinase activity, Lawrence et al. used a fragment-based approach and identified (R)-9b [148], which has proven effective in suppressing the growth of both prostate cancer and triple-negative breast cancer cell lines [149]. (R)-9b potently inhibits Ack1-mediated phosphorylation of Tyr-176 on AKT, though its effects on WASP Ser phosphorylation and other substrates have not yet been reported.

## 7. Regulation of Immune and Inflammatory Pathways

### 7.1. RIPK2 (RIPK2)

Receptor-interacting protein kinase 2 (RIPK2) is a dual-specificity **Ser/Thr and Tyr protein kinase** that plays an essential role in inflammation and innate immunity through the NOD/RIPK2/NF-*κ*B signaling pathway [150,151]. RIPK2 acts downstream of the NOD-like receptors NOD1 and NOD2 (nucleotide-binding oligomerization domains 1 and 2), which together regulate cytokine production in response to intracellular bacterial infection [152,153]. NOD1 and NOD2 bind distinct components of bacterial peptidoglycan and ATP and form a signaling complex with RIPK2. Aberrant NOD/RIPK2 signaling is implicated in the pathogenesis of numerous inflammatory diseases. NOD1 polymorphisms have been linked to susceptibility to early-onset inflammatory bowel disease [154], sarcoidosis [155], and asthma [156]. Specific NOD2 polymorphisms within the leucine-rich repeat (LRR) domain have been linked to Crohn’s disease [157]. The expression levels of both NOD2 and activated/phosphorylated RIPK2 are upregulated in pediatric patients with active Crohn’s disease or ulcerative colitis [158,159]. RIPK2′s involvement in inflammatory diseases makes it a promising drug target. 

RIPK2 contains two domains, an N-terminal catalytic kinase domain with homology to the other RIPK family members and a unique caspase activation/recruitment domain (CARD), which facilitates interactions with NOD1 and NOD2 [160,161]. RIPK2 binding to NOD1 or NOD2 induces its autophosphorylation on residues Tyr-474 and Ser-176 [162,163], leading to Lys-63-linked polyubiquitination at Lys-209 [164]. Subsequent activation of the NF-κB pathway and mitogen-activated protein kinases (MAPKs) thereby induce autophagy [165] and transcription of proinflammatory cytokines and chemokines [166]. Several inhibitors have been found to effectively inhibit RIPK2 kinase activity, with WEHI-345 [167], OD36, and OD38 [168] exhibiting improved specificity. WEHI-345 was found to delay activation of NF-κB on NOD stimulation and prevented cytokine productive both in vitro and in vivo. Interestingly, the crystal structure of FDA-approved ATP-competitive inhibitor ponatinib revealed an allosteric site that may provide an avenue for designing more specific RIPK2 inhibitors [169].

### 7.2. Ser/Thr Protein Kinase 16 (STK16)

Serine-threonine protein kinase 16 (STK16), also known as myristoylated/palmitoylated serine/threonine-protein kinase, is a dual-specificity **Ser/Thr and Tyr protein kinase** with a C-terminal protein kinase domain and N-terminal myristoylation and palmitoylation site at Cys-6 and -8 [170]. It is part of the Numb-associated kinases (NAK) family, with only 25% sequence identity to its closest structural homolog Aurora kinase A [171]. STK16 is constitutively active, which is partially explained by its unique activation segment, which contains a β sheet at the tip of the activation loop and a large α-helical insert [171]. Strikingly, it replaces the conserved DFG motif with a DLG motif, a sequence variation found in less than 6% of human kinases [171].

STK16 directly regulates actin polymerization and depolymerization, thereby controlling the Golgi complex integrity [172]. It enhances the pro-angiogenic capacity of tumors as a transcriptional co-activator of VEGF secretion [173]. It phosphorylates developmentally-regulated GTP-binding protein 1 (DRG1) and 4EBP1 and is involved in the TGF-β signaling pathway, linking it to the regulation of cell growth and homeostasis [171,172]. Discovery of the highly selective STK16 inhibitor STK16-IN-1, which has potent activity both in vitro and in live cells, has provided a useful tool for further investigation of this underexplored kinase and its disease involvement [174].

### 7.3. Lipopolysaccharide Kinase WaaP (rfaP)

WaaP is a bifunctional **sugar kinase and Tyr protein kinase** in *Pseudomonas aeruginosa*, *E. coli*, and *Salmonella* species that is responsible for phosphorylation of the inner core heptose (HepI) of lipopolysaccharide (LPS) [175]. LPS is a major virulence factor for *P. aeruginosa*, an opportunistic Gram-negative pathogen commonly associated with nosocomial infections, pneumonia in cystic fibrosis patients, soft-tissue infections in burn victims, osteomyelitis, sepsis, and ecthyma gangrenosum in immunocompromised patients. *P. aeruginosa* LPS is composed of lipid A endotoxin, a core oligosaccharide divided into an inner and outer core, and O-antigen. The inner oligosaccharide core contains heptose and octulosonic acid and is the most highly phosphorylated among Gram-negative bacteria [176]. LPS contributes to the low outer-membrane permeability of *P. aeruginosa*, which in combination with multiple efflux pumps and production of antibiotic-inactivating genes, confers a high intrinsic antibiotic resistance [177].

WaaP is essential to *P. aeruginosa* viability; knockout is lethal and can only be tolerated if another copy of WaaP is added in trans [175]. The Virulence Factors Database lists WaaP as an essential virulence factor for *P. aeruginosa* pathogenicity [178]. Mutation of WaaP in *Salmonella enterica* serovar Typhimurium causes loss of virulence in vivo and increased antibiotic susceptibility [179]. Inducible WaaP depletion in *P. aeruginosa* blocks core phosphorylation, causing truncation of the LPS core, marked invaginations of the LPS inner membrane, and increased susceptibility to polymyxin B and novobiocin [180].

Although WaaP does not demonstrate true in vivo protein kinase activity, it shares sequence homology with conserved functional residues of Ser/Thr protein kinases protein kinase Cα and SNF1 and Tyr protein kinases Src and EGFR [181]. It autophosphorylates on Tyr residues and phosphorylates exogenous poly(GluTyr) peptide. Its crystal structure revealed structural homology with eukaryotic kinases including an activation loop DFG motif, a catalytic HRD motif, a glycine-rich P-loop, and a catalytically invariant Lys at position 51 [182]. Mutation of structurally predicted essential kinase residues increased the *P. aeruginosa* susceptibility to EDTA or completed the abrogated cellular viability [182], indicating a potential avenue for therapeutic development. WaaP’s structural and functional similarity to eukaryotic protein kinases and its integral role in LPS synthesis and *P. aeruginosa* growth make it a ripe target for existing small molecule protein kinase inhibitors, which may orthogonally inhibit its sugar kinase activity. Disruption of the outer membrane’s stability and the resulting increase in permeability could potentially re-sensitize *P. aeruginosa* to treatment with first-line antibiotics.

## 8. Regulation of Metabolism

### 8.1. PEPCK (PCK1)

Phosphoenolpyruvate carboxykinase (PEPCK) is a **carboxylyase and Ser/Thr protein kinase** that has canonically been described as a gluconeogenic enzyme. PEPCK is the rate-limiting enzyme of gluconeogenesis, where it catalyzes reversible decarboxylation and phosphorylation of oxaloacetate [183]. Its role as a protein kinase in lipogenesis was only recently uncovered. PEPCK phosphorylation at Ser-90 by AKT results in its translocation from the cytosol to the ER, where it transfers a phosphate group from GTP to ER-anchor proteins INSIG1 and -2 on Ser-207 and Ser-151, respectively. INSIG phosphorylation disrupts the binding of cholesterol-derived oxysterols, resulting in activation of sterol regulatory element-binding proteins (SREBPs), transcription of lipogenesis-related genes, and tumorigenesis in mice. Notably, staining of patient-derived hepatocellular carcinoma (HCC) tumors showed a correlation between high levels of phosphorylation at PEPCK1 Ser-90, INSIG1 Ser-207, and INSIG2 Ser-151 with decreased overall durations of survival, linking PEPCK’s alternative catalytic function to the clinical aggressiveness of HCC [184]. Metabolic reprogramming of PEPCK has also been found in pancreatic [185] and esophageal carcinomas [186], warranting further investigation of its role as a protein kinase. GTP-competitive PEPCK inhibitors have previously been developed as anti-diabetic agents [187,188]. Given that GTP serves as the main phosphate donor for PEPCK, these pre-existing inhibitors may also abrogate its protein kinase activity.

### 8.2. Pyruvate Orthophosphate Dikinase Regulatory Protein (PDRP)/Phosphoenolpyruvate Synthetase Regulatory Protein (ppsR)

Pyruvate orthophosphate dikinase regulatory protein (PDRP) is a bifunctional **Ser/Thr protein kinase and pyrophosphorylase** involved in the regulation of gluconeogenesis protein pyruvate orthophosphate dikinase (PPDK). PPDK regenerates phosphoenolpyruvate from pyruvate and ATP in C_4_ plant photosynthesis [189,190]. PDRP phosphorylates Thr-527 on PPDK during inactivation and dephosphorylates it during activation in response to changes in lighting [191]. Although it is primarily found in plants, PDRP also shares sequence homology with the domain of unknown function (DUF)299 genes present in some bacteria, which led to the identification of PDRP homologs in bacteria as well as an analogous protein phosphoenolpyruvate synthetase regulatory protein (PSRP) [192]. PSRP is a bifunctional **Ser/Thr kinase and pyrophosphorylase** involved in the regulation of phosphoenolpyruvate synthetase (PEPS), a critical metabolic switch for bacterial survival on small carbon substrates that catalyzes the phosphorylation of pyruvate to PEP. Based on our UniProtKB search results, PDRP is present in various bacterial species including pathogens *Bacillus anthracis*, *Bacillus cereus*, *Bartonella henselae*, *Clostridium botulinum*, *Rickettsia prowazekii*, *Listeria monocytogenes*, and *Staphylococcus aureus*, whereas PSRP is found primarily in *Escherichia coli*, *Salmonella* species, and *Shigella* species. The bacterial kinases have been insufficiently researched, but the mechanism has been well-characterized in *Arabidopsis thaliana* and *Zea mays.*

PDRP phosphorylates and dephosphorylates PPDK active site Thr-527 in *Zea mays* using ADP as its phosphoryl donor [190,193]. The resulting phosphothreonine residue interferes with the activity of the catalytic His-529 residue. *Zea mays* PDRP forms a homodimer with an N-terminal domain and C-terminal domain that share the same unique protein fold and contain phosphate-binding P-loops [194]. Structural superimposition with *M. pneumoniae* HPrK/P and enzymatic activity assays of mutant PDRP revealed that the C-terminal domain contains a single active site used for both kinase and phosphorylase functions, while the N-terminal domain is proposed to have allosteric regulatory effects. Further characterization of these proteins is necessary given their high prevalence in our search results.

### 8.3. Isocitrate Dehydrogenase Kinase/Phosphatase (aceK)

Isocitrate dehydrogenase kinase/phosphatase (IDHK/P) is a bifunctional **Ser/Thr protein kinase and phosphatase with specific ATPase activity**. It phosphorylates and dephosphorylates isocitrate dehydrogenase, a key enzyme in the Krebs cycle responsible for the conversion of isocitrate to α-ketoglutarate, based on nutrient availability [195,196,197]. It was the first example of prokaryotic protein phosphorylation [198,199] and is only seen in Gram-negative bacteria including *Escherichia coli*, *Klebsiella*, *Pseudomonas*, *Yersinia*, *Shigella*, and *Salmonella* species. It serves as the metabolic switch between the Krebs cycle and the glyoxylate bypass, an anaplerotic pathway that allows net conversion of acetyl-CoA to carbohydrates via isocitrate lyase and malate synthase. This allows bacteria to survive on acetate and fatty acids without the loss of carbon as CO_2_. IDHK/P phosphorylates isocitrate dehydrogenase on Ser-113, completely inactivating it through occlusion of the isocitrate binding site [200].

IDHK/P contains two distinct domains, a catalytic C-terminal domain that contains the kinase, phosphatase, and ATPase functions, and an N-terminal regulatory domain, which contains small binding pockets for modulating the enzymatic activity [199]. The kinase domain resembles eukaryotic Ser/Thr and Tyr protein kinases including the IL-1 receptor and FGFR, while the regulatory domain contains a unique protein fold [201]. The ATP-binding site contains an invariant Lys-336, which is conserved among protein kinases [202]. Loop β3αC is homologous with the eukaryotic activation loop and controls accessibility to this ATP-binding site. Residues 484–510 form a substrate recognition loop (SRL) that is critical for the highly specific binding to isocitrate dehydrogenase homodimers [203]. A kinetic analysis of WT and two mutant IDHK/P proteins indicated that the kinase, phosphatase, and ATPase catalytic functions of the enzyme share the same active site [204,205].

### 8.4. HPr Kinase/Phosphorylase (hprK)

HPr kinase/phosphorylase (HPrK/P) is a bifunctional **Ser/Thr protein kinase and phosphorylase** that is a central sensor for carbon catabolite repression in bacteria. It was first identified as a Ser protein kinase sensitive to cellular concentrations of metabolic intermediates. HPrK/P phosphorylates and dephosphorylates Ser-46 of HPr, a phosphocarrier protein of the phosphoenolpyruvate:carbohydrate phosphotransferase system [206,207,208]. In the presence of glucose, catabolite control protein CcpA forms a ternary complex with Ser(P)-46-HPr and the catabolite responsive element DNA sequence to repress transcription of alternative sugar pathway operons [209,210]. HPrK/P is also linked to the transcriptional regulation of cell adhesion and virulence of Gram-negative pathogens [211]. In *Listeria monocytogenes*, formation of Ser(P)-46-HPr inhibits PrfA, a transcriptional activator of virulence genes including the hemolysin-encoded *hly* [212].

The crystal structures of the HPrK/P catalytic domain from *Lactobacillus casei* and the full-length kinase/phosphorylase from *Mycoplasma pneumoniae* and *Staphlycoccus xylosus* revealed a structure unrelated to eukaryotic protein kinases [213,214,215]. HPrK/P is a homohexamer with a three-fold axis orthogonal to three two-fold axes, with each half of a hexamer subunit containing a central loop [216]. Each subunit contains a Walker A ATP-binding motif forming a typical phosphate-binding loop (P-loop). The C-terminal domain, which contains the P-loop, carries both kinase and phosphorylase activities, and the N-terminal domain is currently poorly understood but hypothesized to have an allosteric regulatory role [216]. Co-crystallization of the *L. casei* HPrK/P catalytic domain and *B. subtilis* HPr demonstrated substrate binding at sites of the hexamer where two subunits overlap. Superposition of the *L. casei* V267F HPrK/P mutant, which adopts the kinase conformation, with WT HPrK/P in the phosphorylase conformation, demonstrated that the same active site catalyzes both enzymatic functions [217]. The HPrK/P catalytic function is allosterically mediated by ATP concentration; the presence of ATP favors the kinase conformation whereas the absence of ATP favors the phosphorylase conformation. Ramstrom and coworkers identified a bacteriostatic inhibitor of B. subtilis HPrK/P, Do9, with micromolar activity against both its kinase and phosphorylase activities [216,218]. Intriguingly, competition experiments with Do9 indicate that it is not ATP-competitive, suggesting an allosteric binding mode that may yield more selective inhibition of microbial growth.

### 8.5. Multidomain Regulatory Protein Rv1364c (Rv1364c)

Rv1364c is a **bifunctional Ser/Thr protein kinase and phosphatase** only found in pathogenic *Mycobacterium tuberculosis.* Bacteria respond to environmental stressors including changes in temperature, oxygen, and osmotic stress by modifying their patterns of gene expression with alternative σ factors, which provide promoter recognition sites for RNA polymerase. σ factors are fine-tuned by anti-σ factors and anti-σ factor antagonists. In *Mycobacterium tuberculosis*, the general stress response σ factor is σ^F^, which is regulated in part by Rv1364c, a unique multi-domain polypeptide only found in pathogenic mycobacteria [219]. In contrast to the homologous multiprotein system present in *Bacillus subtilis*, Rv1364c encodes the entire toolbox for σ^F^ regulation on a single polypeptide. It contains an N-terminal Per-Arnt-Sim (PAS) domain, which senses the energy levels in the cells, followed by a PP2C-like phosphatase domain, a kinase domain, and a sulfate transporter and AntiSigma factor antagonist (STAS) domain, which binds to σ^F^ on phosphorylation [220]. The STAS signaling domain is phosphorylated on Ser-600 by the kinase domain and dephosphorylated by the phosphatase domain [221,222]. Phosphorylation promotes dimerization and suggests a system where phosphorylation-dependent partner-switching of domains controls the bacterial transcriptional response [221]. Misra et al. proposed an orthogonal model in which the eukaryotic-like Ser/Thr kinase protein kinase D serves as an osmotic sensor to phosphorylate Rv1364c and release σ^F^, linking extracellular sensing to the σ^F^ regulon [220]. Further investigation of this virulence factor is necessary given the high worldwide mortality of *M. tuberculosis* and the emergence of drug-resistant strains.

## 9. Discussion

In this review, we sought to identify serine/threonine and tyrosine kinases that harbor additional enzymatic activities. We probed the UniProtKB database for kinases with multiple EC annotations to uncover the frequency at which these dual activities are encoded by the same polypeptide chain. From our findings, we highlight seventeen human kinases and five bacterial kinases that have multiple catalytic activities and that are involved in the regulation of phosphoinositide signaling, transcription/translation, cell growth and differentiation, immunity, and metabolism. The search returned some kinases that are well-established as critical signaling mediators and drivers of human disease, making them promising drug targets. On the other hand, the remaining kinases require further functional characterization. Nine of these bifunctional kinases have separate catalytic functions within the same polypeptide chain, with their functions tightly controlled through upstream signaling effectors and allosteric regulation. The other 13 kinases are dual-specificity protein kinases and nucleoside triphosphatases with promiscuous substrate specificity that (i) may have no biological relevance, or (ii) could be critical for their signaling roles.

There are broad implications for kinases with multiple catalytic activities. For example, how do the evolutionary benefits differ between one protein with a single multifunctional domain (e.g., IDHK/P), one protein with two domains with two distinct functions (e.g., LRRK2), and two proteins with two distinct functions? Both HPrK/P and IDHK/P couple their contrasting kinase and phosphatase activities to the same multifunctional domain, and furthermore, to the same active site. Given that these proteins serve as key bacterial metabolic switches, their bifunctional active sites suggest an evolutionary benefit to harboring exquisite allosteric control. In addition, Rv1364c combines a multi-polypeptide signaling system from *B. subtilis* into a single polypeptide in *M. tuberculosis*. Consolidation of its kinase and phosphatase activities to a single protein may impart a pathogenic advantage by controlling localization to ensure the rapid temporal control of these contrasting activities.

The results in this study only represent 879 of the total 36,160 protein kinases from our search. As we continue to study existing proteins in more detail with more sensitive biochemical, structural, and computational methods, we can expect to identify additional enzymatic activities from well-characterized enzymes. For example, PEPCK, long-known as the rate-limiting enzyme of gluconeogenesis, has recently been identified as a protein kinase in an orthogonal and clinically relevant signaling pathway. Given the other metabolic enzymes identified in our search, this suggests that there are other metabolic kinases with non-canonical protein kinase activity [223].

In our study, we identified bifunctional kinases with dual phosphatase, phosphorylase, endoribonuclease, acetyltransferase, carboxylyase, and lipid/sugar kinase enzymatic activities. Given our findings, it is plausible that proteins within these functional groups could exhibit secondary kinase activities that have yet to be elucidated. Furthermore, it would be illuminating to extend this study to the human proteome and classify all bifunctional enzymes, which could potentially reveal evolutionary benefits of pairing certain enzymatic functions. We theorize that bifunctionality within the kinome and the proteome at large represents an exciting field of study.

## Figures and Tables

**Table 1:** Unique bifunctional kinases in the UniProtKB database.

Kinase (*Gene*)	UniProtKB ID *	Functions	Biological Relevance	Length (Amino Acids) *	Total Number of Species/Strains
**PI3Kα/PI3Kγ/PIKfyve (*PIK3CA/PIK3CG/PIKFYVE*)**	P42336 P48736 Q9Y2I7	Ser/Thr kinase Phosphotransferases with alcohol groups as acceptors	Cell growth and proliferation Tumorigenesis	1068 1102 2098	5 3 3
**Ire1α (*ERN1*)**	O75460	Ser/Thr kinase Endoribonuclease	ER unfolded protein response	977	7
**Ire1β (*ERN2*)**	Q76MJ5	Ser/Thr kinase Endoribonuclease	Translational repression through 28S ribosomal rRNA cleavage	926	3
**Protein kinase R (*EIF2AK2*)**	P19525	Ser/Thr kinase Tyr kinase	Innate immunity to viral infection NF-κβ inflammasome activation Apoptosis Cell proliferation and differentiation	551	3
**RIOK1 (*RIOK1*)**	Q9BRS2	Ser/Thr kinase Hydrolase (ATPase)	40S ribosomal biogenesis	568	10
**Transcription initiation factor TFIID subunit 1 (*TAF1*)**	P21675	Ser/Thr kinase Histone acetyltransferase	Scaffold for TFIID basal transcription factor complex	1872	3
**MHC class II transactivator (*CIITA*)**	P79621	Ser/Thr kinase Histone acetyltransferase	Coactivator of MHC Class II promoter Enhances MHC class I transcription	1155	2
**PRPK (*TP53RK*)**	Q96S44	Ser/Thr kinase Hydrolase (ATPase)	EKC/KEOPS complex subunit responsible for tRNA threonylcarbaymoyl adenosine modification	253	45
**PKCδ (*PKCD*)**	Q05655	Ser/Thr kinase Tyr kinase	Cell cycle progression Apoptosis Tumorigenesis	676	3
**LRRK2 (*LRRK2*)**	Q5S007	Ser/Thr kinase Hydrolase (ATPase)	Neuronal plasticity/neurodegeneration Autophagy Vesicle trafficking	2527	2
**Maternal embryonic leucine zipper kinase (*MELK*)**	Q14680	Ser/Thr kinase Tyr kinase	Cell cycle regulation and proliferation Apoptosis Tumorigenesis	651	2
**ACK1 (*TNK2*)**	Q07912	Tyr kinase Ser/Thr kinase	Cell spreading and migration Proliferation Tumorigenesis	1038	6
**RIPK2 (*RIPK2*)**	O43353	Ser/Thr kinase Tyr kinase	Innate and adaptive immunity	540	3
**Ser/Thr protein kinase 16 (*STK16*)**	O75716	Ser/Thr kinase Tyr kinase	Implicated in actin dynamics Tyr autophosphorylation	305	3
**WaaP (*rfaP*)**	Q9HUF7 (*P. aeruginosa*)	Sugar kinase Tyr kinase	Phosphorylation of the inner core heptose (HepI) of lipopolysaccharide (LPS)	268 (P. aeruginosa)	1
**PEPCK (*PCK1*)**	P35558	Carboxylyase Ser/Thr kinase	Rate-limiting enzyme of gluconeogenesis SREBP activation and lipogenesis	622	7
**PDRP (*PDRP1*)/PSRP (*ppsR*)**	Q195N6 (*Z. mays*) P0A8A4 (*E. coli*)	Ser/Thr kinase Pyrophosphorylase	Regulation of pyruvate orthophosphate dikinase/phosphoenolpyruvate synthetase	426 (Z. mays) 277 (E. coli)	403
**IDHK/P (*aceK*)**	P11071 (*E. coli*)	Ser/Thr kinase Phosphatase ATPase	Sensor for glyoxylate bypass in Krebs cycle	578 (E. coli)	122
**HPrK/P (*hprK*)**	O34483 (*B. subtilis*)	Ser/Thr kinase Phosphorylase	Sensor for carbon catabolite repression	310 (B. subtilis)	214
**Rv1364c (*Rv1364c*)**	P9WLZ7 (*M. tuberculosis*)	Kinase Phosphatase	Osmoregulatory sensor	653 (M. tuberculosis)	2

* Refers to *H. sapiens* unless otherwise indicated.

## Data Availability

Not applicable.

## Supplementary Materials

The following supporting information can be downloaded at: https://www.mdpi.com/article/10.3390/biom12050685/s1. Table S1: Full list of UniProtKB results from search algorithm. Table S2: Manually curated and reviewed UniProtKB results. Table S3: Grouped list of final 22 unique bifunctional kinases across species/strains from the UniProtKB search results.
